# Higher frequency of hamstring injuries in elite track and field athletes who had a previous injury to the ankle - a 17 years observational cohort study

**DOI:** 10.1186/s13047-018-0247-4

**Published:** 2018-02-26

**Authors:** Nikolaos Malliaropoulos, Georgios Bikos, Maria Meke, Korakakis Vasileios, Xavier Valle, Heinz Lohrer, Nicola Maffulli, Nat Padhiar

**Affiliations:** 1 Sports and Exercise Medicine Clinic, Thessaloniki, Greece; 2 National Track & Field Centre, Sports Medicine Clinic, Thessaloniki, Greece; 3 European Sports Care, London, UK; 4 Sports Clinic, Rheumatology Department, Barts Health Trust, London, UK; 5 William Harvey Research Institute, Centre for Sports and Exercise, Barts and The London School of Medicine and Dentistry, Queen Mary, University of London, London, UK; 6 Euromedica Arogi Rehabilitation Clinic, Thessaloniki, Greece; 7 Aspetar, Orthopaedic and Sports Medicine Hospital, Doha, Qatar; 8 Hellenic Orthopaedic Manipulative Therapy Diploma, Athens, Greece; 9 Football Club Barcelona, Medical Department, Barcelona, Spain; 10 Sports Medicine School (Universitat de Barcelona), Barcelona, Spain; 11 Mapfre Centre for Tennis Medicine, Barcelona, Spain; 12 Department de Cirurgia de la Facultat de Medicina at the Universitat Autònoma de Barcelona, Barcelona, Spain; 13 European SportsCare Network, Frankfurt am Main, Germany; 14 Department of Sport and Sport Science, University of Freiburg, Freiburg im Breisgau, Germany; 15 Department of Musculoskeletal Disorders, Faculty of Medicine and Surgery, University of Salerno, Salerno, Italy

**Keywords:** Ankle injuries, Hamstring injuries, Track and field athletes, Sports injuries

## Abstract

**Background:**

Inversion injury to the ankle and hamstring injuries are common problems in most sports. It is not known whether these injuries constitute a predisposing factor or a precursor of injury or re-injury of these anatomical locations. Therefore, we wished to test the hypothesis that a previous inversion ankle injury exerted a significant effect on the chance of an athlete suffering from a subsequent ipsilateral hamstring injury and vice versa.

**Methods:**

In an observational cohort study over 17 years (1998–2015), 367 elite track and field athletes, were grouped according to their first traumatic isolated ankle or hamstring injury. Fifty athletes experienced both injuries. The Mann-Whitney U and Chi-square tests (*p* < 0.05) were performed to test possible associations of ankle and hamstring injury with age, gender, athletics discipline, grade, and type of antecedent injury.

**Results:**

Athletes with a preceding ankle injury had a statistically significantly higher chance of experiencing a subsequent hamstring injury compared with athletes who had experienced a hamstring injury as their first traumatic event (x^2^ = 4.245, *p* = 0.039). The proportion of both ankle and hamstring injury events was not statistically different between female (18%) and male (11%) athletes. Age and grade of injury did not influence the proportion of ankle and/or hamstring injury events.

**Conclusion:**

There is a statistically significantly higher frequency of hamstring injuries in elite track and field athletes having experienced a previous ankle ligament injury.

## What is known about the subject?

To our knowledge, this is the first study that assessed the association between ankle and hamstring injuries and their predisposing role that each one of them has in a re-injury affecting the other location.

## What this study adds to existing knowledge

The present study opens the way to investigate the relationship between injuries occurring in different parts of the musculoskeletal system but in the same limb.

Athletes with a previous ankle injury face a higher risk of hamstring injury.

In clinical practice, there is a need for a holistic approach to musculoskeletal rehabilitation which should not be directed only to the region where a given injury occurred.

## Introduction

Ankle sprains and hamstring muscle injuries are among the most common sport injuries, and are a major cause of time lost from sport participation [1–5].

The main mechanisms of hamstring injury is eccentric contraction at high velocity and slow stretching at outer range of motion [6]. A hamstring injury is a major risk factor for future hamstring injury [7–10]. Lateral ankle sprains are usually provoked by excessive foot inversion with the foot in plantar flexion [11]. Certain somatometric factors such as higher longitudinal foot arch, wider foot, cavovarus deformities of the foot may be associated with this injury [12]. Additionally, there is an association of the incidence of lateral ankle injuries with reduced cardiorespiratory endurance, decreased muscle strength and range of motion of ankle dosriflexors and decreased movement coordination [13].

There are several extrinsic risk factors for lower limb injuries such as level of competition, skill level, shoe type, ankle bracing and playing surface [14]. Intrinsic risk factors include age, gender, limb dominance, limb flexibility, body size, foot morphology, anatomical alignment of the lower limb, muscle tightness, joint laxity, aerobic fitness, postural stability and inadequate rehabilitation after previous musculoskeletal injury [14].

In addition, a recent systematic review concluded that a single ankle sprain is the most common injury resulting in a secondary sprain of the ipsi- or contralateral ankle [15].

Furthermore, a growing body of evidence links proximal and distal contributors/risk factors to lower extremity injury [16]. A pathomechanical model has been proposed, linking the biomechanics of the ankle joint with development of patellofemoral pain syndrome [17, 18], altered position and function of the hip and pelvis [19, 20] and the development of low back pain [21]. According to this model, excessive foot pronation delays external rotation of the tibia and disrupts timing between knee extension and rearfoot supination [18, 22, 23].

A link has also been suggested in terms of the role of proximal structures in biomechanical function of the lower limb and the development of lower extremity injury [24–26].

The core muscles (lumbar-pelvic hip complex) are essential in controlling hip abduction, subsequent femoral internal rotation, and potentially more distal movement [17, 27, 28].

There is nevertheless a gap in the literature evaluating whether a prior injury in the lower limb could be a predisposing factor for an injury occurring in a different location in the same lower limb.

This study compared and evaluated the events of first time unilateral hamstring and ankle injuries in elite track & field athletes over a period of 17 years, and focused concurrently on both these injuries and their possible pathophysiological interdependence, while the majority of the studies reports on each of these injuries in isolation.

## Methods

### Study design

First-time traumatic ankle ligament injuries and hamstring muscle injuries in elite track & field athletes were assessed through medical records at the Sports Medicine Clinic of National Team Elite Track & Field Centre of Northern Greece in Thessaloniki. All injuries were recorded for a study period of 17 years (1998 to 2015) via a non-probability purposive sampling method. The Elite Track & Field Centre and Sports Medicine Clinic, where data were collected, was the only centre that National Team elite track and field athletes were attending in the case of an injury. Weekly meetings with the national team coaches allowed the identification and follow up of each injured athlete and therefore, it was very unlikely for the sports medicine team not to be informed of any injury. The study was approved by the National Track & Field Federation.

**Fig. 1:**
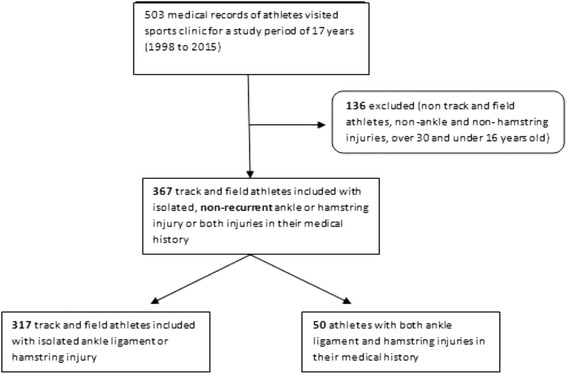
Flowchart of the study population

Finally, a total of 367 elite track & field athletes aged 16 to 30 years, having visited the Sports Medicine Clinic of the National Team Elite Track & Field Centre, were included. Relatively injured athletes were excluded if the injury was not related to ankle ligament or hamstrings, and if they participated in sports other than track and field (Fig. 1).

The athletes were grouped into four categories related to sport disciplines: sprinters (100 m, 200 m, 400 m, 110 m), throwers (hammer, javelin, discus, shotput), jumpers (pole vault, long, triple and high jumps) and combined events athletes (decathlon, heptathlon).

**Fig. 2:**
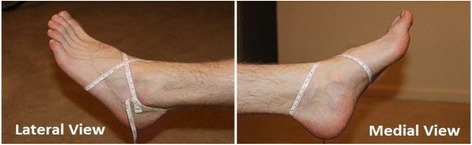
Measurement of oedema in ankle injuries

Ankle injuries were classified in four grades according to Malliaropoulos et al. [29, 30] (Table 1).The measurement of oedema was performed using a measuring tape so that the following landmarks were crossed in a figure-of-eight fashion (Fig. 2): a) navicular tuberosity, b) the distal tip of the lateral malleolus, c) the distal tip of the medial malleolus and d) the base of the fifth metatarsal. The measurement was compared with the uninjured ankle, and it was expressed as oedema difference. Stress radiographs [29, 30] were performed in patients with a grade II and III injury with the knee positioned at 45 degrees of flexion and the foot in neutral to 5 degrees of plantar flexion. A 5 kg (Kg) weight was hanging from the lower third of the tibia [30].

**Table 1:** Revised criteria for the classification of acute injury of the lateral ligament complex of the ankle compared to the uninjured side. Malliaropoulos et al. Foot Ankle Clinics 2006 Sep; 11(3):497–507

Grade	Decreased ROM	Edema difference	Stress radiographs
I	Up to 5 degrees	Up to 0.5 cm	Normal
II	5 to 10 degrees	0.5 cm to 2 cm	Normal
IIIA (III)	More than 10 degrees	More than 2 cm	Normal
IIIB (IV)	More than 10 degrees	more than 2 cm	Laxity greater than 3 mm

In the hamstring injuries’ group of athletes, the inclusion and exclusion criteria are described in Table 2 [31].

**Table 2:** Inclusion and exclusion criteria for the hamstring injury group (Malliaropoulos et al. Br J Sports Med 2014; 48:22 1607–1612)

Inclusion criteria	Exclusion criteria
Acute injury	Uncertain clinical diagnosis
Local tenderness	Verified or previously suspected posterior thigh muscle injury
Pain with resisted knee flexion or resisted hip extension	Pain on palpation at the origin or insertion of the posterior thigh muscles
Pain with passive hip flexion with the knee extended	
Provocation of pain on isometric contraction of posterior thigh muscles	Extrinsic trauma to the posterior thigh or bilateral injuries Tendon avulsion or total rupture of hamstring muscles Chronic low back pain and/or sciatica

Hamstring injuries were classified in four grades according to Malliaropoulos et al. [32, 33] (Table 3).

**Table 3:** Classification of posterior thigh muscle injury according to active range of motion deficit of knee extension. Malliaropoulos et al. Am J Sports Med. 2010 Sep; 38(9):1813–9

Clinical grade	Active ROM deficit
I	Up to 9 degrees
II	10 to 19 degrees
III	20 to 29 degrees
IV	More than 30 degrees

Clinical assessment was conducted by a single certified fully trained Sport and Exercise physician, and included a detailed medical history (symptoms related to their sport, mechanism of the injury), a thorough clinical examination (including observation of stance and gait, palpation, range of motion, muscle strength testing and special diagnostic tests). Additionally, if required according to Ottawa ankle rules, athletes underwent diagnostic ultrasound and ankle radiographs [34].

### Study population

During the data collection period, 367 elite track and field athletes, 225 (61.3%) males and 142 (38.7%) females, visited the Clinic reporting acute traumatic ankle and/or hamstring injuries. The athletes’ mean age at their first visit was 20 ± 3.2 years (range 13 to 34).

Not reported athletic disciplines in elite track and field athletes’ medical records were treated as elite track and field athletes’ with missing discipline. A total of 144 runners, 99 jumpers, 19 throwers, 25 combined events athletes and 80 elite track and field athletes with missing information on their event discipline, were included in the study (Table 4).

**Table 4:** Population sport events and gender

			Athletic discipline					Total
			Runners	Jumpers	Throwers	Combined	Elite Track & Field athletes (missing discipline)	
Gender	Male	Number	81	67	12	14	51	225
		% within Gender	36.0%	29.8%	5.3%	6.2%	22.7%	100.0%
		% within Athletic discipline	56.2%	67.7%	63.2%	56.0%	63.8%	61.3%
		% of Total	22.1%	18.3%	3.3%	3.8%	13.9%	61.3%
	Female	Number	63	32	7	11	29	142
		% within Gender	44.4%	22.5%	4.9%	7.7%	20.4%	100.0%
		% within Athletic discipline	43.8%	32.3%	36.8%	44.0%	36.2%	38.7%
		% of Total	17.2%	8.7%	1.9%	3.0%	7.9%	38.7%
Total		Number	144	99	19	25	80	367
		% within Gender	39.2%	27.0%	5.2%	6.8%	21.8%	100.0%
		% within Athletic discipline	100.0%	100.0%	100.0%	100.0%	100.0%	100.0%
		% of Total	39.2%	27.0%	5.2%	6.8%	21.8%	100.0%

## Statistical analysis

Initially, all track and field athletes included were grouped in two categories according to the first traumatic event occurring in one of the two sites under study (ankle injury or hamstring injury). The athletes were then grouped in two categories indicating the occurrence of a traumatic event in both sites or in one of the sites over the time of the study. Frequencies and proportions were calculated and correlated with age, gender, athletic discipline and grade of injury, in both cases and based on the total of the 367 athletes having been injured either in one or in both sites under study.

The risk of the elite track and field athletes under study to experience either a hamstring or an ankle injury was calculated as an incidence proportion. Consequently, the numerator includes only new cases of hamstring or ankle injuries, whereas the denominator is the number of elite track and field athletes having been recorded and followed up during the observation period.

Therefore, the incidence proportion was estimated as follows: [35].

[(Number of events per type of injury) / (total of athletes during a specified period and registration terrain)*100].

The Mann-Whitney U test was performed to assess possible associations between continuous and categorical data. The Chi-square test was performed to test possible associations between categorical variables. The Monte Carlo method was used in both cases to estimate the significance level. If there was a 2 × 2 table, exact results were provided instead of Monte Carlo. The basic assumption that 0 cells (0.0%) should have expected a count less than 5 was accepted. Statistical analysis was conducted with Stata 12.0 statistical software and confidence level was set at 0.05.

## Results

There was a higher incidence of hamstring injuries (16.3%, 245 incidents) compared with ankle injuries (8.1%,122 incidents). The proportion of athletes having experienced both injuries accounted for 3.3% (50 incidents out of 1500 athletes) of the total number of athletes with any type of injury.

**Fig. 3:**
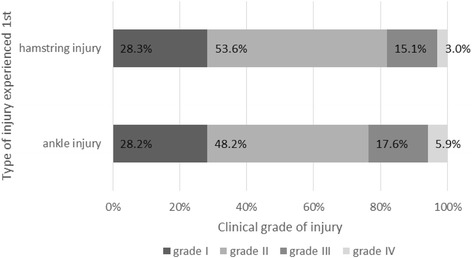
Proportion according to grades of ankle and hamstring injury

Hamstring injuries experienced as first event accounted for 66.8% (245 athletes) and ankle injuries accounted for 33.2% (122 athletes), considering that there was no antecedent injury in these two areas. Most ankle injuries were clinical grade II (48.2%), followed by grade I (24%), and grade III (17.6%). Similarly, a grade II injury was recorded in 53.6% of the hamstring injuries, followed by grade I (28.3%) and grade III (15.1%) (Fig. 3).

Age was not statistically significantly different between ankle and hamstring injuries reported as first injury (U = 7674, *p* = 0.692). Additionally, gender was not statistically significantly associated with the type of first injury recorded (× 2 = 3.492, *p* = 0.324).

**Fig. 4:**
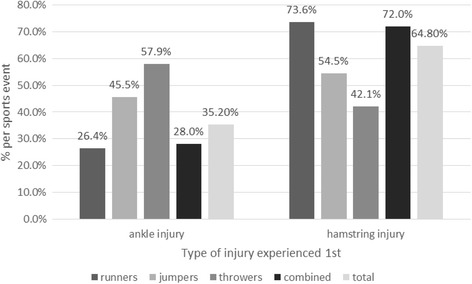
Frequency of ankle and hamstrings injury in the study population

The athletic discipline was statistically significantly associated with the type of first injury (× 2 = 14.325, *p* = 0.002). Hamstring injuries were significantly more frequent than ankle injuries, especially in runners, jumpers, and combined sports events: 73.6% of the runners, 54.5% of the jumpers, and 72% of the combined events athletes experienced a hamstring injury as first injury (Fig. 4).

**Fig. 5:**
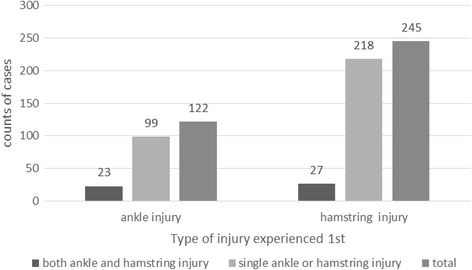
Frequency of single ankle or hamstring injury, and ankle and hamstring injury

From a total of 367 elite track and field athletes who had experienced a first injury of the hamstring or of the ankle, 13.6% (50 athletes out of 367 athletes) experienced both injuries over the data collection period (Fig. 5). Athletes with a preceding ankle injury (23/122 = 19%) had a higher chance of experiencing a subsequent hamstring injury compared with athletes who had experienced a hamstring injury as the first traumatic event (27/245 = 11%; × 2 = 4.245, *p* = 0.039).

Athletic discipline (× 2 = 3.025, *p* = 0.388) and gender (× 2 = 3.120, *p* = 0.77) did not significantly affect the number of athletes experiencing both ankle and hamstring injuries. The proportion of athletes experiencing both ankle and hamstring injury events was 18% (25 of 142) in females and 11% (25 of 225) in male athletes. The clinical grade of ankle or hamstring injury did not influence the subsequent traumatic event to occur in the different area (× 2 = 7.633, *p* = 0.54).

## Discussion

To our knowledge, this is the first study that assessed the interdependence of acute traumatic ankle and hamstring injuries and their predisposing role in a re-injury affecting the other location. The most important finding of this study was the fact that athletes with a previous ankle injury had higher proportion of a subsequent first injury in the hamstrings compared with athletes with a previous hamstring injury and a subsequent ankle injury (*p* < 0.05). Also, athletes who first suffered from an ankle injury were at greater risk of further ankle injuries. This study does not answer the question, why a hamstring injury following ankle injury is more common than an ankle injury following hamstring injury. This interesting finding has to be investigated in further prospective research.

Generally, injury rates increase with the number of preceding injuries [36]. Furthermore, psychosocial factors such as risk-taking behaviour, life event stress and trait anxiety may contribute to an increased risk of re-injury even in a different location [37, 38].

Lower extremity injuries are multifactorial [39], and depend on several intrinsic (athlete-related) and extrinsic (environmental) factors. In some instances, intrinsic factors have proven more predictive of muscle strain injury than extrinsic factors [40]. In the present study, the frequency of both ankle and hamstring injury events was greater in female (18%) than in male (11%) athletes, although the difference was not statistically significantly. Interestingly, age did not influence the proportion of ankle and/or hamstring injury events.

Up to now, the literature has not associated directly ankle and hamstring injury, even though there is evidence to suggest that previous calf muscle injury is independently predictive of hamstring muscle injury. After injury, changes may occur in the functional biomechanics of the lower limb, predisposing the athletes to injury in different muscle groups. Accordingly, altered lower limb biomechanics have been reported in gait, unilateral stance and functional testing after ankle sprain injuries compared with controls [40].

A significant increase in ankle joint inversion and a reduction in joint plantar flexion during gait have been found in patients following lateral ankle sprain [41]. Limited ankle dorsiflexion has also been proposed as a predictor for lateral ankle sprain [42]. Kinetic and kinematic alterations were observed, and were attributed to an increased reliance on more proximal structures (knee and hip) to absorb impact forces [41]. Patients with a 6 month history of first-time lateral ankle sprain displayed a greater hip-dominant coordination strategy for static unilateral stance [43] and greater hip extensor dominance during drop jumps [44]. The observed flexor movement produced during the initial phase of the drop jumps may have led to a force attenuation strategy following initial contact [44]. A plausible explanation could be given for the findings of the present study by the fact that 32–74% of individuals with a history of ankle sprain report residual and chronic symptoms and aberrancy of sensorimotor variables of neuromuscular control [45–47]. This situation alters lower limb biomechanics in the injured ankle and affects proximal muscle groups such as hamstrings [48]. Previous injury in the lower extremity, especially when followed by inadequate rehabilitation, is a risk factor for further injuries to the ankle, knee, and to the ipsilateral lower extremity in general [14].

Regarding athletes with a prior history of acute ankle injury, neurophysiological changes have been reported in the ipsilateral posterior thigh muscles [49–51]. Additionally, mechanical instability of the ankle joint leads to an increased inversion, predisposing to further ankle sprain injury [52].

Evidence suggests at least an indirect biomechanical link between specific regions of the leg. Tight hamstring muscles have been associated with plantar fasciitis [45, 48, 53]. Increased hamstring tightness causes early contraction of the posterior leg muscles through the gait cycle, and decreases ankle dorsiflexion [54, 55] which in turn induces prolonged forefoot loading and the increased magnitude of tensile loading forces within the plantar fascia [45].

There is need for further studies on the interdependence of injuries in these specific anatomic sites. The outcome of the current study underlines the need for consequent rehabilitation of ankle and hamstring muscle injuries. Further research should address the interferences between hamstring and ankle injuries. In addition, it seems reasonable to elucidate, if the observed injury associations also occur in different sports (e.g. football). The question, if the demonstrated relations are also true for recreational athletes has also to be answered in future investigations. Probably, there is also an influence of a specific injury to induce a contralateral lesion.

## Conclusion

The study population of elite track and field athletes showed a significantly higher frequency of hamstring injuries if an athlete had sustained a prior traumatic acute ankle injury compared with athletes who initially had posterior thigh muscle injury and secondarily suffered an ankle joint injury. Given the present findings, rehabilitation programmes should focus on improvement of proprioception and strength of the whole lower extremity after ankle injuries.

In addition, the association between injuries occurring in different parts of the musculoskeletal system needs to be investigated further.

## Limitations

One limitation of the study was the fact that 80 athletes did not report their sport discipline. However, their injuries were recorded in detail, and therefore it does not bear any consequence on the main study question.

Recall bias regarding correct reporting of first injury was also likely to be present during the follow up period. Also, the grading of severity of the two injuries is not homogeneous, so, for example, a grade II ankle injury is not comparable, in terms of severity and recovery time, to a grade II hamstring injury. Another potential confounder was the heterogeneity of the study population. Although all included subjects were elite track and field athletes, they were divided in different sport disciplines with different load demands to the lower extremities.

## Abbreviations

Kg: Kilogram
UK: United Kingdom
